# Supplementary material for: WordCommentsAnalyzer: A windows software tool for qualitative research
Source: F1000Res. 2018 Sep 4;7:536. Originally published 2018 May 3. [Version 2] doi: 10.12688/f1000research.14819.2 (PMC6137411; doi:10.12688/f1000research.14819.2)
Supplement: Supplementary file 3 [file f1000research-7-17614-s0002.tgz › b2a79496-4ada-47c4-9682-a99ce867836d.zip!/Eight Iranian Health Journals/Yafteh _2.docx]

© Yafteh  . This work is licensed under CC BY 4.0 (https://creativecommons.org/licenses/by/4.0/).

Volume & Issue: دوره 19 - شماره 4

Title: تحلیل محتوایی و استنادی مجله علمی-پژوهشی یافته دانشگاه علوم پزشکی لرستان طی سال های 1390 تا 1394

http://yafte.lums.ac.ir/article-1-2454-fa.html

مقدمه: باتوجه به اهمیت تحلیل محتوایی و استنادی مجلات در شناخت کیفیت علمی مجلات، هدف از مطالعه حاضر تحلیل محتوایی و استنادی مجله علمی-پژوهشی یافته دانشگاه علوم پزشکی لرستان طی سال­ های 1390 تا 1394 بوده است. مواد و روش‌ها: این پژوهش یک مطالعه طولی گذشته نگر است که در سال 1395 انجام شد، مقالات منتشر شده در بین سال­های 1390 تا 1394 مورد بررسی قرار گرفتند. داده­ها با استفاده از یک چک لیست محقق ساخته  جمع ­آوری و با استفاده از نرم افزار SPSS نسخه 16 و آمار توصیفی، تجزیه و تحلیل شدند. یافته ­ها: یافته­ ها نشان داد که 8/95 درصد از مقالات چاپ شده به صورت اصیل بوده­اند و بیشترین وابستگی سازمانی نویسندگان مسئول، مربوط به دانشگاه علوم پزشکی لرستان (8/53 درصد) بوده است. در طی پنج سال اخیر 262 مقاله در مجله یافته چاپ شده است. نیمه عمر منابع 38/8 سال بوده و بیشترین شماره در سال ­های 91 و 92 چاپ شده ­اند. بحث و نتیجه ­گیری: با توجه به نتایج بدست آمده از پژوهش حاضر پیشنهاد می­شود که مسئولین مجله در جهت برقراری تعادل در وابستگی سازمانی مقالات منتشر شده، اقدامات لازم را به عمل آورند. همچنین با تعیین محدودیت ­هایی، نویسندگان را به استفاده از منابع جدیدتر ترغیب نمایند.

Title: بررسی و مقایسه اثر 8 هفته تمرینات استقامتی و تناوبی شدید بر بیان ژن گیرنده‌های ایکس کبدی (LXR) در رت‌های نر نژاد ویستار

http://yafte.lums.ac.ir/article-1-2468-fa.html

مقدمه: پژوهش حاضر با هدف تعیین تأثیر تمرینات استقامتی و تناوبی شدید بر بیان ژن گیرنده‌های کبدی در رت‌های نر نژاد ویستار انجام‌ شده است. مواد و روش‌ها: روش تحقیق تجربی بوده و به همین منظور تعداد 24 سر رت نر نژاد ویستار با محدوده وزنی 20±200 گرم و سن هشت هفته، تهیه و به‌صورت تصادفی به سه گروه کنترل، تمرین تناوبی شدید و تمرین تداومی زیر بیشینه تقسیم شدند. پروتکل تمرینی خیلی شدید؛ 30 دقیقه دویدن تناوبی (هر تناوب شامل 4 دقیقه دویدن با شدت 90-85 درصد VO2max و 2 دقیقه بازیافت فعال با شدت 60-50 درصد VO2max) پنج روز در هفته و به مدت 8 هفته اجرا شد. همچنین گروه تمرین تداومی زیر بیشینه نیز شدت فعالیتی معادل 50 تا 55 درصد حداکثر اکسیژن مصرفی موش‌ها بود. یافته ­ها: بیان ژن LXR آلفا، در تمرین تناوبی شدید افزایش معناداری به نسبت گروه کنترل داشت (004/0P=). همچنین اختلاف معناداری بین 3 گروه در میزان بیان ژن LXR  بتا وجود ندارد (001/0P=). هرچند نتایج نشان از افزایش جزئی در گروه‌های تمرین تناوبی و تداومی بود اما این افزایش اندک منجر به ایجاد تفاوت معنادار نشده است. بحث و نتیجه ­گیری: تمرین تناوبی شدید به نسبت تمرینات تداومی زیر بیشینه از طریق افزایش بیان ژن گیرنده مهم کبدی و همچنین عامل اصلی خروج کلسترول از کبد و درنهایت گیرنده HDL می‌تواند نقش مهمی در کاهش بیماری‌های قلبی-عروقی مانند آترواسکلروزیس داشته باشد.

Title: پیش بینی اضطراب امتحان و خودناتوان سازی تحصیلی براساس الکسی تایمیا

http://yafte.lums.ac.ir/article-1-2394-fa.html

مقدمه: هدف این مطالعه پیش ­بینی اضطراب امتحان و خود ناتوان­سازی تحصیلی براساس الکسی ­تایمیا در دانش ­آموزان با ناتوانی خواندن بود. مواد و روش­ها: این مطالعه از نوع همبستگی است. جامعه آماری تمامی دانش ­آموزان با ناتوانی خواندن شهرستان خرم­ آباد در سال 94-95 بود. به منظور برآورد حجم نمونه از روش پیشنهادی کوهن استفاده شد و از میان دانش­ آموزان ارجاع داده شده به هسته آموزش و پرورش خرم­آباد براساس تشخیص متخصصان این حوزه، در مجموع 112 نفر واجد ملا ک ­های شرکت در مطالعه بودند. دانش ­آموزان پرسشنامه ­های اضطراب امتحان، خود ناتوان­ سازی و الکسی­ تایمیا را تکمیل کردند. برای تحلیل داده ­ها از تحلیل رگرسیون استفاده شد. داده­ ها با استفاده از نرم افزار آماری spss نسخه 18 تحلیل شدند. یافته ­ها: نتایج نشان داد همبستگی بین متغیرهای پژوهش معنادار بود (p>0.001). همچنین نتایج تحلیل رگرسیون نشان داد الکسی­ تایمیا قادر به پیش­بینی 54/.  از کل واریانس اضطراب امتحان و خودناتوان­سازی تحصیلی است(001/0>P).

Title: فراوانی دیابت پنهان خودایمن در بیماران با فنوتیپ دیابت نوع 2 در قم

http://yafte.lums.ac.ir/article-1-2376-fa.html

مقدمه: دیابت پنهان اتوایمیون بزرگسالی (Latent autoimmune diabetes of adults) نوعی دیابت است که پیشرفت کندی داشته و قبل از نیازمندی به انسولین با رژیم غذایی و داروهای کاهنده قند خوراکی کنترل می شود. از سنجش اتوآنتی بادیها جهت تشخیص افتراقی LADA و دیابت نوع2 استفاده می شود. هدف طرح بررسی فراوانی بیماران LADA در بین مبتلایان به دیابت نوع  2 با استفاده از سنجش اتوآنتی بادی های ضد جزیره و ارزیابی ویژگی های کلینیکی و بیوشیمیایی  آنها بود. مواد و روش‌ها: در این مطالعه توصیفی – مقطعی 86 بیمار دیابت نوع 2 با محدوده سنی 28 تا 67 سال جهت بررسی حضور اتوانتی بادی های ضد جزیره با روش الایزا ارزیابی شدند. برای تشخیص افراد LADA آنتی بادی های ضد گلوتامیک اسید دکربوکسیلاز، سلول های جزیره و انسولینوما آنتی ژن2 ارزیابی شدند. یافته­ ها: از 86 بیمار با فنوتیپ دیابت نوع 2 تعداد 35 نفر (70/40درصد) از نظر آنتی بادی مثبت و 51 نفر (30/59 درصد) برای هر نوع آنتی بادی منفی بودند. بین دو گروه از نظر سن در هنگام مطالعه، درمان با انسولین، سطوح پپتید C و سابقه بیماری های خودایمنی اختلاف معنی داری مشاهده شد. از طرفی اختلاف معنی داری بین دو گروه از نظر سایر فاکتورهای خطر وجود نداشت. بحث و نتیجه ­گیری: در بین بیماران دیابت نوع 2، تعداد بیماران دیابت پنهان خودایمن بالا بوده و همچنین تعداد افرادی که آنتی بادی چندگانه دارند شیوع بالایی دارد.

Title: تأثیر مکمل سازی کوتاه مدت آب گریپ فروت بر پراکسیداسیون لیپیدی و ظرفیت آنتی اکسیدانی تام پلاسما و برخی شاخص های آسیب عضلانی مردان جوان متعاقب یک جلسه فعالیت مقاومتی شدید

http://yafte.lums.ac.ir/article-1-2390-fa.html

مقدمه: نتایج برخی داده های علمی تأثیرات مثبت آنتی اکسیدان های طبیعی را بر تعدیل شاخص های ظرفیت آنتی اکسیدانی تام و آسیب عضلانی گزارش کرده اند. پژوهش حاضر با هدف بررسی مکمل سازی کوتاه مدت آب گریپ فروت بر پراکسیداسیون لیپیدی و ظرفیت آنتی اکسیدانی تام پلاسما و برخی شاخص های آسیب عضلانی مردان جوان متعاقب یک جلسه فعالیت مقاومتی شدید انجام شد. مواد و روش‌ها: در یک کارآزمایی نیمه تجربی دو سویه کور، تعداد 20 دانشجو پسر انتخاب و به طور تصادفی در 2 گروه، دارونما و مصرف کننده آب گریپ فروت قرار گرفتند. آزمودنی ها پس از دوره ی مکمل سازی یک وهله فعالیت حاد مقاومتی با شدت 85 درصد یک تکرار بیشینه را انجام دادند. تغییرات ظرفیت آنتی اکسیدانی تام، مالون دی آلدئید، کراتین کیناز و لاکتات دهیدروژناز طی سه مرحله اندازه گیری شد. یافته­ ها: در مطالعه حاضر مشاهده شد که تغییرات ظرفیت آنتی اکسیدانی تام و مالون دی آلدئید پلاسمای گروه مصرف کننده آب گریپ فروت پس از فعالیت مقاومتی بر خلاف گروه دارونما معنی دار نبود. همچنین غلظت کراتین کیناز و لاکتات دهیدروژناز پلاسمای هر دو گروه پس از فعالیت مقاومتی افزایش معنی داری نشان داد.  نتیجه گیری: مصرف آب گریپ فروت ظرفیت آنتی اکسیدانی تام پلاسما را ارتقاء داده و از افزایش معنی دار مالون دی آلدئید پلاسما متعاقب فعالیت مقاومتی جلوگیری کرده و منجر به تعدیل افزایش سطوح کراتین کیناز تام و لاکتات دهیدروژناز می شود.

Title: تعیین میزان آگاهی پزشکان عمومی از بیماری هپاتیت E در شهر خرم آباد

http://yafte.lums.ac.ir/article-1-2166-fa.html

مقدمه: هپاتیت E نوع بیماری عفونی ویروسی است که کبد را درگیر می کند و می تواند از یک عفونت ساده بدون علامت تا یک بیماری برق آسا و کشنده بخصوص در زنان باردار، متغیر باشد. هدف مطالعه اخیر بررسی سطح آگاهی پزشکان عمومی شهر خرم­آباد در مورد بیماری هپاتیت E بوده است. مواد و روش‌ها: این مطالعه یک مطالعه اپیدمیولوژیک توصیفی است که سطح آگاهی پزشکان عمومی در دسترس شهر خرم­آباد در مورد بیماری هپاتیت E مورد ارزیابی قرار می گیرد. اطلاعات مورد نظر در قالب پرسشنامه گردآوری و با روش­های آمار توصیفی نظیر جداول توزیع فراوانی، جداول توافقی، میانگین ، انحراف معیار و نسبت مورد تجزیه و تحلیل قرار گرفت. یافته­ ها:در مطالعه حاضر 100 پزشک عمومی مورد بررسی قرار گرفتند که 62 نفر را مردان و 38 نفر را زنان تشکیل می­دهند. در مطالعه ما برای بررسی سطح آگاهی پزشکان عمومی شهر خرم آباد در مورد بیماری هپاتیت E یک پرسشنامه ی 14 سوالی طراحی گردید. میزان آگاهی در خصوص راههای انتقال بیماری و میزان شیوع بیماری ضعیف اما در سایر جنبه های بیماری متوسط بود. تفاوت آماری معنی داری بین سطح آگاهی پزشکان زن و پزشکان مرد نبود. اما در حیطه راههای کنترل و پیشگیری نمره اخذ شده توسط پزشکان زن بالاتر بود. نمره کلی آگاهی در پزشکان با سابقه کاری زیر پنج سال تفاوت آماری معناداری نسبت به پزشکان با سابقه کاری بالای 15 سال داشت. بحث و نتیجه­گیری: سطح آگاهی کلی پزشکان عمومی شهر خرم آباد در مورد بیماری هپاتیت E متوسط می باشد که با توجه به شیوع حدود 8 درصدی در شهر خرم آباد و میزان مرگ و میر بالا در زنان باردار و جوانان، آموزش بیشتر مورد نیاز است.

Title: بررسی اثرات دیکلوفناک و ایبوپروفن بر تکثیر سلول های نرمال کلیوی HEKدر محیط کشت سلولی

http://yafte.lums.ac.ir/article-1-2323-fa.html

مقدمه: مطالعات نشان می دهند که داروها بر روی سلول های نرمال بدن اثرگذار هستند. هدف اصلی این تحقیق، بررسی اثرات دیکلوفناک و ایبوپروفن بر زنده مانی سلول های نرمال کلیویHEK  در محیط کشت انتخابی بوده است. مواد و روش‌ها: در این مطالعه تجربی-آزمایشگاهی سلول های HEK به طور تصادفی به گروه شاهد و گروه های تحت تاثیر دوزهای  001/0،  01/0، 1/0، 1، 10 میلی گرم بر میلی لیتر داروهای دیکلوفناک و ایبوپروفن، تقسیم شدند. سپس مقدار اثر سمیت داروها با استفاده از سنجش MTT  مورد سنجش قرار گرفت. داده ها با استفاده از روش آماری آزمونی واریانس یک طرفه بین گروه ها مقایسه شدند. یافته ­ها: زنده مانی سلول های HEK در معرض دوزهای 001/0، 01/0، 1/0 میلی گرم/ میلی لیتر داروی ایبوپروفن در مقایسه با گروه شاهد دچار افزایش غیر معنادار شده است و زنده مانی سلول های HEK در گروه های دریافت کننده دوزهای 001/0، 01/0، 1/0، 1، 10 میلی گرم بر میلی لیتر از داروی دیکلوفناک در مقایسه با گروه شاهد دچار کاهش معنا دارشده است. (به ترتیب 01/0P<،  05/0P<، 01/0P<، 01/0P<، 01/0P<). بحث و نتیجه ­گیری: دارو های دیکلوفناک و ایبوپروفن در دوزهای بالا باعث از بین رفتن سلول های HEK می شوند. بنابراین استفاده از این دارو ها در دوزهای بالا نامناسب است.

Title: بررسی شیوه ی زندگی متأثر از فضای مجازی و تاثیر آن بر سلامت روان (مطالعه موردی دانشجویان دانشگاه علوم پزشکی لرستان در سال1394)

http://yafte.lums.ac.ir/article-1-2342-fa.html

مقدمه: گسترش سریع رسانه ­های جمعی و به ویژه فضای مجازی باعث تغییر در شیوه­ی زندگی مردم شده است. شیوه­ی زندگی متأثر از فضای مجازی از عواملی هست که بر سلامتی افراد تأثیرگذار می­ باشد. لذا در این مطالعه به بررسی شیوه­ی زندگی متأثر از فضای مجازی و تاثیر آن بر سلامت روان پرداخته شد. مواد و روش‌ها: روش پژوهش، توصیفی-پیمایشی از نوع هدف کاربردی می­باشد جامعه آماری شامل کلیه دانشجویان دانشگاه علوم پزشکی لرستان در سال تحصیلی 95-1394 می­باشد. برای نمونه گیری با فرمول کوکران، 361 نفر با نمونه گیری تصادفی طبق­ای انتخاب گردید. داده­ها با پرسشنامه مشتمل بر سه بخش مشخصات جمعیت شناختی، شیوه­ی زندگی متاثر از فضای مجازی طاهری و سلامت روان گلدبرگ، جمع­آوری گردید. تحلیل داده ­ها در دو بخش آمار توصیفی و استنباطی با استفاده از نرم­افزار SPSS19 و Amos انجام شد. یافته­ ها: میانگین نمره شیوه­ی زندگی متاثر از فضای مجازی بر حسب جنسیت، پایگاه اجتماعی-اقتصادی و وضعیت تأهل متفاوت نبود. همچنین رابطه معنادار و مستقیم بین شیوه­ی زندگی متاثر از فضای مجازی و سلامت روان وجود داشت. مدل معادله ساختاری نیز، حاکی از  تأیید داده­ های گردآوری شده با چارچوب نظری بود. بحث و نتیجه ­گیری: با توجه به ارتباط بین شیوه­ی زندگی متاثر از فضای مجازی و سلامت روان، توصیه می­شود که مسئولین برنامه ­هایی را اتخاذ نمایند که موجب تقویت، گسترش و مدیریت رسانه ­های جمعی نوین شوند و به این ترتیب در افزایش سلامت روان دانشجویان تأثیر گذار باشند.

Title: تاثیر 6 ماه تمرین هوازی با شدت متوسط بر سطوح BDNF، IL-6 و حافظه کوتاه مدت زنان 50- 65 ساله‌ی مبتلا به سندروم متابولیک

http://yafte.lums.ac.ir/article-1-2267-fa.html

مقدمه: افزایش سن، عوامل التهابی و سندروم متابولیک از عوامل موثر در کاهش BDNF و عملکرد شناختی می‌باشد. هدف از این تحقیق بررسی اثرگذاری 6 ماه تمرین هوازی با شدت متوسط بر سطوح BDNF،  IL-6و حافظه کوتاه مدت زنان 65-50 ساله مبتلا به سندروم متابولیک می‌باشد. مواد و روش‌ها: 24 زن مبتلا به سندروم متابولیک به طور داوطلبانه در این تحقیق شرکت کردند. آزمودنی ها به طور تصادفی به دو گروه 12 نفره ی مبتلا به سندروم متابولیک تمرین (ME) و کنترل (MC) تقسیم شدند. گروه تمرین، در یک برنامه تمرینی هوازی شش ماهه که هر هفته سه جلسه، هرجلسه شامل سه ست تمرینی و دو ست استراحت بود شرکت کردند. در ابتدا مدت زمان ست تمرینی 8 دقیقه بود که با سپری شدن هر هفته تا هفته دوازدهم، یک دقیقه به مدت زمات ست­های تمرینی افزوده می­شد. خونگیری در دو مرحله پیش و پس آزمون جهت اندازه گیری سطوح BDNF و IL-6 انجام شد. میزان سطوح BDNF و IL-6 توسط رادیوایمونواسی و میزان حافظه کوتاه مدت نیز توسط آزمون یادداری عددی مورد بررسی قرار گرفت. یافته­ ها:  BDNF و حافظه کوتاه مدت پس از 6 ماه تمرین هوازی نسبت به پیش آزمون افزایش معنی‌داری کردند (05/0P˂). همچنین IL-6 پس از 6 ماه تمرین هوازی نسبت به پیش آزمون کاهش معنی‌داری داشت (05/0P˂). بحث و نتیجه ­گیری: در اثر تمرین هوازی 6 ماهه با شدت متوسط، میزان سطوح IL-6 کاهش و BDNF افزایش یافت که در نتیجه این فرایند موجب بهبود حافظه کوتاه مدت گردید.

Title: اثر محافظتی عصاره دارچین در برابر آسیب های کبدی القا شده توسط ایبوپروفن در موش های صحرایی

http://yafte.lums.ac.ir/article-1-2333-fa.html

مقدمه: ایبوپروفن از خانواده داروهای ضد التهاب غیراستروئیدی و مسکن درد است. در عین حال این دارو دارای اثرات ثابت شده ای مانند تولید رادیکالهای آزاد و تداخل با وقایع سلولی می باشد.  بنابراین یافتن آنتی اکسیدان های مناسب به منظور کاهش عوارض جانبی این دارو  ضروری است. در این تحقیق اثر آنتی اکسیدانی عصاره دارچین  بر مهار آسیب کبدی ناشی از ایبوپروفن بررسی شده است. مواد و روش‌ ها: 48 رت در 6 گروه، گروه S نرمال سالین دریافت کردند، گروه  C200 عصاره دارچین با دوز 200 میلی گرم به  ازای هر کیلوگرم وزن بدن (mg/kg200)، گروه  Iایبوپروفنmg/kg 400، گروه  IC50ایبوپروفن به همراه عصاره دارچین mg/kg 50، گروه IC100  ایبوپروفن به همراه عصاره دارچین mg/kg 100 و گروه IC200  ایبوپروفن همراه عصاره دارچین mg/kg 200 بصورت داخل صفاقی دریافت کردند. در پایان دوره فعالیت آنزیم های کبدی اندازه گیری شد. از کبدها نمونه بافتی تهیه شد و پس از رنگ آمیزی با هماتوکسیلین - ائوزین، مورد مطالعه قرار گرفتند.    یافته ­ها: فعالیت آنزیم­های پیروات ترانس آمیناز، اگزالواستات ترانس آمیناز و آلکالین فسفاتاز در گروه  Iنسبت به گروه S بطور معناداری افزایش داشتند. در گروه های  IC50،IC100  و IC200  تقریباً  متناسب  با دوز عصاره دارچین نسبت  به گروه I میزان فعالیت آنزیم ها کاهش داشتند. مطالعه برش بافتی کبد حاکی از تخریب بافتی ناشی از ایبوپروفن بود که  مصرف دارچین تخریب بافتی را  کاهش داده بود. بحث و نتیجه ­گیری: عصاره دارچین باخواص آنتی اکسیدانی خود آسیب کبدی ناشی از مصرف ایبوپروفن را کاهش می دهد.

Title: بررسی ارتباط بین هوش هیجانی و گرایش به اعتیاد در دانشجویان دانشگاه علوم پزشکی لرستان

http://yafte.lums.ac.ir/article-1-2449-fa.html

مقدمه: هوش هیجانی نوعی پردازش هیجانی اطلاعات است که می تواند باعث محافظت فرد در برابر مصرف مواد شود. پژوهش حاضر با هدف بررسی ارتباط بین هوش هیجانی و گرایش به اعتیاد در دانشجویان دانشگاه علوم پزشکی لرستان انجام شد.   مواد و روش‌ها: در این پژوهش توصیفی–تحلیلی تعداد 223 نفر از دانشجویان دانشگاه علوم پزشکی لرستان به روش نمونه گیری تصادفی خوشه ای انتخاب شدند.اطلاعات از طریق پرسشنامه های اطلاعات دموگرافیک ، گرایش به اعتیاد (APS) و هوش هیجانی  (berry-graves)جمع آوری و با استفاده از آزمون های آنالیز واریانس، آزمون t و ضریب همبستگی پیرسون مورد تجزیه و تحلیل آماری قرار گرفتند. یافته ­ها: میانگین امتیاز گرایش به مصرف مواد در دانشجویان 47/28  و میانگین امتیاز هوش هیجانی 72/81 بود.یافته های این پژوهش نشان داد که بین هوش هیجانی و گرایش به اعتیاد ارتباط منفی معناداری وجود دارد(05/0P<).  همچنین بر اساس نتایج پیرسون، بین گرایش به اعتیاد و خرده آزمون های خودآگاهی ،خود مدیریتی ،آگاهی اجتماعی و مدیریت رابطه ،ارتباط معناداری وجود دارد(05/0P<). بحث و نتیجه ­گیری: افراد با هوش هیجانی پایین تر ،تمایل بیشتری به سوء مصرف مواد دارند. برنامه های آموزش هوش هیجانی می تواند عامل پیش گیرنده در اعتیاد محسوب شود.

Title: بررسی میزان شیوع شکایات اختلالات اسکلتی عضلانی در کارمندان بخش اداری دانشگاه علوم پزشکی شهر خرم آباد در سال 1394

http://yafte.lums.ac.ir/article-1-2384-fa.html

مقدمه: اختلالات اسکلتی- عضلانی عامل اصلی از دست رفتن زمان کار، افزایش هزینه ها و آسیب های انسانی است. لذا بررسی شیوع این اختلالات در بین کارکنان اداری با توجه به مدت زمان کار با کامپیوتر و چگونگی استقرار آنها در پشت میز کار از اهمیت ویژه ای برخوردار است.  مواد و روش‌ ها: این پژوهش بصورت مقطعی بر روی کارمندان بخش اداری دانشگاه علوم پزشکی شهرخرم آباد انجام شد. جهت گردآوری اطلاعات علاوه بر مشخصات فردی و اطلاعات دموگرافیک و  سؤالاتی در مورد متوسط مدت زمان استفاده از کامپیوتر در طول روز، ارزیابی اختلالات اسکلتی-عضلانی کارکنان با استفاده از پرسشنامه استاندارد نوردیک  صورت گرفت. برای تجزیه و تحلیل داده ها از آمار توصیفی برای محاسبه میزان، میانگین و انحراف معیار هر یک از متغیرهای تحقیق و برای بررسی رابطه بین متغیرها از آزمون استقلال مجذور کای استفاده شد. کلیه آنالیزها به کمک نرم افزار 21SPSS  انجام گرفته است. یافته ­ها: نتایج مطالعه نشان داد که بیشترین شیوع علائم اختلالات اسکلتی–عضلانی به  ترتیب در نواحی کمر (60 درصد)، گردن (4/59 درصد)، شانه (50 درصد) بوده و زانو (4/49 درصد)، پشت (1/47 درصد)، مچ دست (4/42 درصد) در رتبه های بعدی قرار دارند. بین سابقه کار و شیوع علائم در نواحی شانه ، زانو ، ران، قوزک پا و گردن در نمونه های مورد مطالعه ارتباط معنی داری وجود دارد (05/0P < ). اما در نواحی آرنج، مچ دست، پشت و کمر، رابطه معناداری مشاهده نشد.  بین سن کارکنان  و شیوع اختلال در ناحیه آرنج  رابطه معنی داری وجود داشت (05/0P < )، به گونه ای که با افزایش سن فقط شیوع اختلال در این ناحیه  افزایش یافته است و در سایر نواحی بدن اختلالی مشاهده نشده است. بحث و نتیجه ­گیری: بر اساس نتایج مطالعه حاضر، اختلالات اسکلتی– عضلانی دارای فراوانی بالایی در بین کارکنان اداری  است. در این راستا انجام برنامه های مداخله ای ارگونومی در محیط کار امری  ضروری بنظر می­رسد. که می توان به آموزش کاربران در رابطه با اصول  ارگونومیک کار با کامپیوتر، طراحی ایستگاه کار و نوبت کاری را به عنوان اجزا این برنامه ذکر کرد.

Title: تاثیر ده هفته تمرینات مقاومتی بر سطوح استراحتی برخی عوامل آنژیوژنز در مردان مبتلا به سرطان پروستات

http://yafte.lums.ac.ir/article-1-2581-fa.html

مقدمه: سرطان پروستات دومین سرطان در مردان و هشتمین علت مرگ در ایران می باشد. در شرایط پاتولوژیک رگزایی باعث افزایش متاستاز و گسترش این سرطان می شود. هدف این تحقیق بررسی تآثیر ده هفته تمرین مقاومتی بر برخی عوامل آنژیوژنز در مردان مبتلا به سرطان پروستات بود. مواد و روش‌ها: 20 مرد مبتلا به سرطان پروستات با دامنه سنی 55 تا 62  سال انتخاب و به صورت تصادفی به دوگروه کنترل ( 10 نفر) و مقاومتی(10 نفر)تقسیم شدند.گروه تجربی به مدت 10 هفته تمرین و هر هفته سه جلسه تمرین مقاومتی را انجام دادند. هر تمرین سه دور با دوازده تکرار و با شدت 50 تا 65 درصد یک تکرار بیشینه انجام شد. 48 ساعت قبل و  48 ساعت بعد از اجرای پروتکل تمرین و پس از 12 ساعت ناشتا نمونه خونی جهت اندازه گیری عامل رشد اندوتلیالی عروق (VEGF )، عامل رشد فیبروبلاست (FGF ) و نیتریک اکساید (NO ) گرفته شد. تغییرات درون  گروهی و بین گروهی به ترتیب با استفاده از آزمون تی  وابسته و تی مستقل مورد تجزیه وتحلیل قرار گرفت. یافته ­ها: نتایج نشان داد انجام 10 هفته تمرین مقاومتی باعث افزایش معنی داری در سطوح  VEGF(0001/0P=)، FGF (0001/0P=) و NO (0003/0P=)، در گروه تجربی نسبت به گروه کنترل در مردان مبتلا به سرطان پروستات شد. بحث و نتیجه ­گیری: به نظر می رسد 10 هفته تمرین مقاومتی باعث افزایش عوامل آنژیوژنز، قدرت و توده عضلانی و کاهش خستگی در بیماران مبتلا به سرطان پروستات می گردد.
